# Gan-du-qing attenuates PM_2.5_-induced Chronic Airway Inflammation via regulating the pulmonary microbiota and metabolic profiles

**DOI:** 10.3389/fmed.2025.1560225

**Published:** 2025-09-10

**Authors:** Yongcan Wu, Biao Zuo, Xin Zhou, Sijing Zhao, Caixia Pei, Xiaomin Wang, Yilan Wang, Demei Huang, Shihua Shi, Zherui Shen, Jianwei Wang, Fei Wang, Zhenxing Wang

**Affiliations:** ^1^Chongqing University of Traditional Chinese Medicine, Chongqing, China; ^2^Chongqing Traditional Chinese Medicine Hospital, Chongqing, China; ^3^Hospital of Chengdu University of Traditional Chinese Medicine, Chengdu, Sichuan, China

**Keywords:** PM_2.5_, chronic airway inflammation, Gan-du-qing, lung microbiota, serum metabolome

## Abstract

**Background:**

Substantial evidence links fine particulate matter (PM_2.5_) to the development of inflammatory lung diseases such as chronic airways, but effective treatments are lacking. Gan-du-qing (GDQ) Decoction is a traditional Chinese medicine formula for chronic airway inflammation. However, whether GDQ can ameliorate PM_2.5_-induced lung injury and its mechanism are unknown, and we will further investigate.

**Study design/Methods:**

Male Sprague-Dawley (SD) rats weighing 120 grams were utilized to establish a rat model of lung injury through systemic exposure to PM_2.5_. We built a real environmental exposure chamber with an exposure period of 16 weeks and the average concentration exposed was 110.5 μg/mł. The exposure chamber is located at 12 Bridge Road, Chengdu City, and the exposure time is from November to February of the second year. GDQ was administered via gavage starting 4 weeks post-exposure. Morphological changes were observed through Hematoxylin and Eosin (HE) staining. Inflammatory cell infiltration was detected using immunohistochemical staining, while scanning electron microscopy was employed to observe ultrastructural changes in the lung trachea. Levels of inflammatory cytokines in bronchoalveolar lavage fluid were quantified using Enzyme-Linked Immunosorbent Assay (ELISA). The main components of GDQ were identified through Ultra-High-Performance Liquid Chromatography-High-Resolution Mass Spectrometry (UHPLC-HRMS). Additionally, a combination of serum metabolomics and 16S gene sequencing of lung microbiota was employed to pinpoint key targets mediating the therapeutic effects of GDQ in the treatment of PM_2.5_-induced lung injury.

**Results:**

The findings indicated that GDQ had the capability to reduce the pathological changes of lung tissue and mitigate inflammatory exudation in the lungs. 16S rRNA gene sequencing revealed that GDQ effectively reduced the richness and diversity of the pulmonary microbiome induced by PM_2.5_ and restored the overall structure of the pulmonary microbiome. Metabolomic analysis identified 65 potential differential metabolites that may contribute to GDQ’s attenuation of PM_2.5_-induced lung injury. These metabolites were mainly enriched in the Phospholipase D signaling pathway, Metabolism of xenobiotics by cytochrome P450, and Glutathione metabolism.

**Conclusion:**

Our research offers valuable insights into how GDQ operates to mitigate PM_2.5_-induced lung injury through the modulation of lung microbiota and serum metabolome. These findings may have important implications for the development of effective strategies to protect against lung injury caused by PM_2.5_.

## 1 Introduction

Fine particulate matter (PM_2.5_) in ambient air has become a significant contributor to air pollution in recent times. A growing body of evidence suggests that exposure to components of PM_2.5_ in ambient air pollution can lead to or worsen lung diseases such as asthma (1), chronic obstructive pulmonary disease (COPD) (2, 3), bronchitis (4), and idiopathic pulmonary fibrosis (5). Therefore, it is crucial to explore the precise impact of PM_2.5_ on lung diseases, especially in areas with high levels of air pollution. The incidence of these diseases is also higher in contaminated areas (6), emphasizing the crucial character of PM_2.5_ in the progression or worsening of underlying lung diseases. Lungs are the organs that first encounter PM_2.5_ particles in the human body and can communicate directly with the outside world (7). Consequently, pulmonary functions can be compromised due to continuous exposure to PM_2.5_. Research has shown that fine particle deposition in the lungs can lead to a significant inflammatory response and a decrease in pulmonary function (8, 9).

Recent advances in high-throughput sequencing technology have challenged the traditional view of lungs as sterile organs, revealing instead a diverse and dynamic microbial community (10, 11). The pulmonary microbiota is crucial in maintaining lung health, playing a vital role in preserving respiratory physiology and immune homeostasis (12). Commensal pulmonary microbiota protects against invasion by foreign or pathogenic microorganisms through various mechanisms, including space-occupying effects, nutrient competition, and secretion of bacteriostatic or bactericidal substances (13). However, in the presence of disease, the microbiota structure can be altered, leading to microecological imbalance (14, 15). Limited research has examined the effects of particulate matter on respiratory microbes. In one study, Li et al. (16) demonstrated that exposure to biofuels and motor vehicle exhaust altered lung microbial and immune dynamics in rats. In human volunteer studies, exposure to PM_2.5_ was found to have a profound impact on respiratory microbiota, with sputum bacterial load and microbiota profiles associated with respiratory function profiles (17). Intratracheal instillation of PM_2.5_ into mice lungs was shown to not only significantly alter the composition of the microbiota but also perturb metabolites involved in various metabolic pathways (18, 19). There is a growing body of evidence connecting exposure to PM_2.5_ with the onset of metabolic disorders (20–22), which may be a crucial link between environmental factors and the increased incidence of respiratory diseases. Metabolic disorders affect about 20% of the world’s population, and their incidence continues to rise rapidly (23, 24). Hence, it is imperative to explore the impact of inhaling ambient PM_2.5_ on the host’s microbiota and the resultant metabolic alterations.

Gan-du-qing (GDQ) is a widely used clinical formula developed by our group specifically for treating PM_2.5_-induced lung injury. This formula has received approval from the China Food and Drug Administration (No.: 2016L05320) for its efficacy in addressing this condition (25, 26). Through a comprehensive transformation process, GDQ has been developed into a compound traditional Chinese medicine that integrates the principles of invigorating qi and detoxification with insights from modern pharmacological research, clinical practice experiences, and advanced pharmaceutical processes. GDQ, the compound traditional Chinese medicine, is comprised of *Astragalus mongholicus* Bunge and *Rhizoma Belamcandae*. Extensive research has demonstrated the effectiveness of GDQ in alleviating pulmonary inflammation by enhancing vital energy, detoxifying the body, and eliminating pathogenic factors. Furthermore, studies have shown that GDQ can effectively improve chronic bronchitis by suppressing the inflammatory response, regulating intestinal flora, and modulating immunity. In summary, GDQ is a highly valued clinical formula that has been rigorously developed and authorized for the management of lung injury induced by PM_2.5_ (25). Its transformative journey into a compound traditional Chinese medicine reflects the integration of traditional knowledge with modern scientific advancements. The therapeutic benefits of GDQ, derived from *Astragalus mongholicus* Bunge and *Rhizoma Belamcandae*, encompass its ability to alleviate pulmonary inflammation, strengthen vital energy, and regulate the immune system while addressing chronic bronchitis (27). Despite its proven therapeutic efficacy in clinical settings, the exact mechanism underlying GDQ’s mitigation of lung injury is not yet fully understood, largely due to the intricate composition of traditional Chinese medicine compounds. Hence, additional research is required to clarify GDQ’s mechanism of action concerning lung injury.

In this investigation, male SD rats were subjected to either filtered air (FA) or ambient PM_2.5_ for a period of 3 months, employing an authentic environmental PM_2.5_ exposure system. The main objective was to examine the influence of PM_2.5_ exposure on the lung microflora and serum metabolism of rats, as well as to evaluate the effects of GDQ on lung flora and serum metabolites. To our knowledge, this is the initial investigation into the impact of traditional Chinese medicine (TCM) on serum metabolic changes and their association with lung microbiota in rats exposed to ambient PM_2.5_ throughout their entire bodies. This study employed a non-targeted metabolomics approach to achieve this goal. The outcomes of this investigation are anticipated to advance our understanding of the potential mechanisms responsible for PM_2.5_-induced metabolic disturbances. Furthermore, they are expected to provide a fundamental framework for future research into the prevention and treatment of PM_2.5_-induced lung injuries using traditional Chinese medicine (TCM).

## 2 Material and method

### 2.1 Animals

We acquired male Sprague-Dawley rats, aged 6 weeks and weighing 90–110 g, from Chengdu Dashuo Experimental Animal Co., Ltd in Chengdu, China. The rats were kept in standard housing conditions, adhering to the guidelines provided by the Animal Experimental Centre. The research project was adhered to the guidelines established by the Experimental Animal Research Ethics Committee at Chengdu University of Traditional Chinese Medicine. Ethical approval for these experiments was granted under the reference number 2022-43.

### 2.2 Whole-body inhalation exposure to real environmental PM_2.5_

In line with the methodology described in our prior research, a real environmental PM_2.5_ exposure system was established (28). Initially, air samples were obtained from the environment through a negative pressure pump and catheter device. A PM_2.5_ detector (DT-9881M) was used to validate the PM_2.5_ content in the air, which indicated high levels of PM_2.5_. Subsequently, the air containing PM_2.5_ was directed into two separate rooms: an air filter control room and a PM_2.5_ exposure room. To effectively eliminate PM_2.5_ from the air, a 3-layer filter was installed at the entrance of the air filtration control room. However, these filters were not implemented in the PM_2.5_ exposure chamber. A monitoring system was put in place in both rooms to maintain uniform environmental conditions, including temperature (22–24 °C), humidity (40%–60%), pressure (18–24 PA), ventilation frequency (20–22/h), and air velocity (0.18 m/s). To maintain a consistent PM_2.5_ concentration within the exposure chamber, a 47 mm Teflon filter with a constant airflow of 0.18 L/min was used.

### 2.3 Experimental protocols

After 1 week of acclimation to the feeding environment, all rats were subjected to the PM_2.5_ exposure system. The rats were randomly allocated to either the AF exposure chamber or the PM_2.5_ exposure chamber, where they were exposed to a 7-day cycle of continuous 24-h exposure for a duration of 16 weeks. For detailed experimental procedures, please refer to Supplementary Material 1.

### 2.4 Preparation of GDQ

Gan-du-qing is composed of two botanicals: *Astragalus mongholicus* Bunge (Huangqi) 20 g and *Rhizoma Belamcandae* (shegan) 10 g. The mass ratio of *Astragalus* to *Radix Astragali* is 2:1. Both botanicals were sourced from the herbal pharmacy of Chengdu University of TCM Hospital. Initially, the two herbs were immersed in pure water at a volume eight times greater for a duration of 1 h, and then they were decocted three times for 30 min each. The three decoctions were combined, filtered, and left to stand, and the supernatant was gathered. The supernatant was then concentrated to a concentration of 1.0 g/mL at 60 °C. The concentrate was frozen at −80 °C for 12 h, followed by evacuation at −30 °C for 48 h, and finally dried at 30 °C for 24 h to obtain the lyophilized powder. It should be noted that 10 g of the original herb is equivalent to 1 g of lyophilized powder. Based on the results of our preliminary clinical study, the recommended daily dose of GDQ lyophilized powder for adults is 0.016 g/kg. The daily dosage for rats was determined at 0.105 g/kg by applying a conversion factor of 6.3 for surface area equivalence between rats and humans (29).

### 2.5 UPLC-MS/MS analysis of GDQ decoction components

To analyze the chemical constituents in GDQ liquid, UPLC-HRMS (Ultra-performance liquid chromatography-high resolution mass spectrometry) was employed. A total of 600 μL of GDQ concentrate sample was taken and mixed with 400 μL of methanol by vortexing. From this mixture, 200 μL was diluted with 200 μL of a 40% methanol aqueous solution. The resulting mixture was vortexed and then centrifuged at 16,000 *g* for 15 min at 4 °C. The supernatant was collected for further analysis. The GDQ extracts were analyzed using a Vanquish UHPLC system (Thermo Scientific, Waltham, MA, USA) equipped with an HSS-T3 column (100 × 2.1 mm, 1.8 μm particle size; Waters) at a column oven temperature of 35 °C. The mobile phase A consisted of water with 0.1% formic acid, while the mobile phase B was acetonitrile with 0.1% formic acid (both solvents were of LC-MS grade and from Fisher chemical). The samples were separated at a flow rate of 0.3 mL/min using the following gradient: 5% B for 1 min, a linear increase to 98% B within 16 min, returning to 5% B in 0.5 min, and then maintaining isocratic conditions at 5% B for 2.5 min.

A Q-Exactive HFX mass spectrometer (Thermo Fisher Scientific, Bremen, Germany) was utilized in combination with the UHPLC system for the analysis. Mass spectrometry acquisition was carried out in both electrospray ionization (ESI) positive and negative modes. Data-dependent acquisition (DDA) mode was employed, wherein the top 10 MS1 ions were selected for obtaining MS/MS spectra. Collision energies (CEs) were set to normalized energy levels of 20, 40, and 60 using a stepwise approach. The data acquisition range spanned from m/z 90 to 1300. The spray voltages used were 3800 (for positive mode) and −3000 (for negative mode), with a sheath gas flow rate of 45. The capillary temperature was maintained at 320 °C, and the probe heater temperature was set to 370 °C.

For liquid chromatography-high-resolution mass spectrometry (LC-HRMS) analysis, it is suggested to perform pre-separation of metabolite pools to distinguish between polar and non-polar metabolites. This step could facilitate easier metabolite identification and matching. In our current study, we employed a broad-spectrum LC-HRMS approach to capture a wide range of metabolites.

### 2.6 Sample collection

Rat lung tissue and bronchoalveolar lavage fluid (BALF) were obtained following previously described methods (27). Rats were first anesthetized, and an incision was made in the anterior cervical skin to expose the left and right main bronchi. The right main bronchus was ligated using sutures, and a 5 mL syringe was used to inject 2 mL of phosphate-buffered saline (PBS) into the left lung. The left lung was gently shaken, and the injected PBS was subsequently aspirated back into the syringe. The collected PBS was centrifuged at 3000 *g* for 5 min at 4 °C, and the resulting supernatant was collected. Additionally, the right lung’s middle lobe was subjected to fixation using 4% paraformaldehyde and subsequently underwent staining utilizing hematoxylin and eosin (H&E). The remaining lung tissue was stored at −80 °C.

### 2.7 Measurement of cytokine levels

Lung tissues was retrieved to quantify inflammatory cytokines. The levels of IL-6, TNF-α, IL-10, and IL-1β in Lung tissues were assessed using ELISA kits obtained from MULTI SCIENCES (Hangzhou, China). According to the manufacturer’s guidelines, the absorbance values of the standard samples were measured, and a standard curve was established by correlating the absorbance values and the corresponding known concentrations of the standard samples. Based on the provided standard curve, the concentrations of the unknown samples were determined through calculation.

Chip detection was performed according to the reagent kit (MILLIPLEX^®^ Analyst 5.1) and standard operating procedures, measuring a total of 10 cytokines in BALF. Each antibody on the chip was tested in quadruplicate. The scanned chip images were analyzed using GenePix Pro 6.0 software to extract raw data, including fluorescence signals and background. During data analysis, the mean of the four technical replicates was calculated for each cytokine, which was then used as the signal value. The signal values were normalized across samples using a positive reference, and the normalized data were subsequently used for concentration quantification.

### 2.8 Identification of Th17/Treg cells using flow cytometry

Seven fresh samples of lung tissues and mesenteric lymph nodes (MLNs) from every group were collected and positioned in RPMI 1640 so chilled containers. To adjust cell density to 10^6^ cells per milliliter, lung tissues and MLNs underwent chopping, filtration using a 300 mesh filter cloth, and the elimination of tissue cell remnants. Draw out 100 μL of cells into a sanitized EP tube, introduce 1 μL of Anti-Rt CD4eBiosciencesTM FITC and Anti-Mo CD25eBiosciencesTM PE antibodies into every EP tube, blend thoroughly, and apply a stain for 30 min at 4 °C to shield them from light. Then wash the tube with 1 mL PBS, centrifuge the tube and discard the supernatant. After stabilizing the cells and subsequently rupturing the membranes, 1 μL of anti-Mo/Rat/IL-17A eBio¬scienceTM Percp-cy5.5 and Alexa Fluor 647 anti-rat FoxP3 Bioleged antibodies were introduced as per the guidelines, thoroughly blended, and dyed at 4 °C for half an hour in lucifugal position. To the mixture, 1 mL of PBS (PH = 7.4) at 4 °C was introduced, thoroughly blended, spun in a 300 *g* centrifuge for 5 min, and discarded the supernatant; then, 400 μL of PBS solution was added, thoroughly mixed, identified using a Cyto-FLEX flow cytometer, and examined with Kaluza 2.1 software.

### 2.9 Analysis via Western blotting

The total proteins found in lungs of seven rats per group were gathered using a radioimmunoprecipitation assay (RIPA), and their concentration was measured with a Bromocresol Green with Albumin (BCA) kit. Total proteins underwent a separation process using sodium twelve-acetate-polyacrylamide gel electrophoresis (SDS - PAGE) gels, followed by their transfer to polyvinylidene fluoride membranes. Membranes underwent blocking with 5% fat-free milk for 2 h at room temperature, followed by an incubation night at 4 °C with primary antibodies ROR-γt (1:1000), Foxp3 (1:1000), GAPDH (1:10000). Proteins underwent incubation with their respective secondary antibodies for a duration of 2 h at ambient temperature. Visualization of the immunoblots was achieved through advanced chemiluminescence (Thermo Fisher Scientific), and their comparative protein levels were assessed via a gel imaging device. Protein levels were determined using the Image PRO Plus 6.0 software, developed by the NIH in Bethesda, MD, USA.

### 2.10 Assessment of pulmonary function

At the conclusion of the experiment, lung function was assessed using the forced oscillation technique (FOT). Lung function tests were performed on rats in each group. Prior to the tests, the rats were anesthetized with a 1% sodium pentobarbital solution. The anterior neck skin was incised to expose the trachea. A minor tracheal incision was created, and a Y-shaped plastic tube was inserted, firmly secured with sutures. Following this, the rat’s airway was connected to a ventilator (AniRes2005 Version 3.5) via the Y-shaped tube. Following the configuration of the program, the ventilator automatically monitored and recorded the lung function parameters of the rats.

### 2.11 Histopathological staining of lung tissue

Fresh lung tissue samples were fixed and embedded in paraffin. The paraffin-embedded lung tissue was subsequently cut into sections that were 5 mm thick for hematoxylin and eosin (H&E) staining. These lung sections were observed under a light microscope by three pathologists. The pathologists evaluated the lung sections and assigned a pathological score using a previously established scoring scale for assessing lung injury (30).

### 2.12 Immunohistochemical (IHC) staining of lung tissue

The lung tissue sections were subjected to dewaxing and washing processes, followed by antigen retrieval using citric acid repair buffer. Subsequently, the sections were blocked with 3% BSA at room temperature for 30 min. We acquired the Anti-Galectin 3 (BS-20700R) antibody and the Anti-Neutrophil (BS-6982R) Elastase antibody from Bioss (Beijing, China). Additionally, the secondary antibodies were procured from Thermo Fisher Scientific (Waltham, MA, USA). The sections were incubated overnight at 4 °C with an anti-galectin-3 antibody (diluted at 1:400) and a neutrophil antibody (diluted at 1:500). After washing the primary antibody, the sections were incubated with HRP-conjugated secondary antibody at room temperature for 50 min. After washing, sections were stained sequentially with 3,3′-diaminobenzidine (DAB) chromogenic solution and hematoxylin. Finally, the sections were dehydrated and fixed. Sections were observed microscopically and assessed quantitatively by Image-pro Plus 6.0 software.

### 2.13 Scanning electron microscopy of lung tissue

Fresh lung tissue blocks were fixed with fixative at room temperature for 2 h and then transferred to a 4 °C refrigerator for storage. Next, the tissue blocks were washed three times with phosphate buffer (pH 7.4) for 15 min each. Then, these tissue blocks were fixed with 1% osmium tetroxide at room temperature in the dark for 2 h. After washing again, the tissue blocks were dehydrated with alcohol and isoamyl acetate. These lung tissue blocks are attached to metallic stubs and sputter-coated with gold for the 30 s. Finally, we observed lung tissue by scanning electron microscopy and collected images.

### 2.14 16S rRNA gene sequencing for the lung microbiome

Bronchoalveolar lavage fluid were collected from rats. The lung microbiome was analyzed using 16S rRNA high-throughput sequencing to investigate the impact of GDQ. The sequencing assay was conducted at Novo-gene Biotechnology (Beijing, China). In brief, total bacterial DNA was first extracted using CTAB. The purity and concentration of the DNA were then detected by agarose gel electrophoresis experiments. The DNA sample was then diluted to 1 ng/μL with sterile water as the sample to be tested. DNA templates are amplified using specific primers with barcodes. The V4 region of the bacterial 16S rRNA gene was used as the amplification region. The sequences of the primer genes used are as follows: 515 forward: GTGCCAGCMGCGGTAA, 806 reverse: GGACTACHVGGGTWTCTAAT.

The library construction was performed using the TruSeq^®^ DNA Free PCR Sample Preparation Kit. The constructed libraries were quantified by Qubit and Q-PCR. After the library is qualified, NovaSeq6000 is used to perform sequencing. The Illumina NovaSeq sequencing platform was used to perform paired-end sequencing of the libraries. The obtained reads were clustered into Operational Taxonomic Units (OTUs) for species annotation and abundance analysis. The species composition and community structure differences between samples were assessed using α-Diversity and β-Diversity measures.

### 2.15 Serum metabonomics analysis

To begin the sample preparation, measure 100 μL of the sample and transfer it to an EP tube. Add 400 μL of an 80% methanol-water solution, and then vortex and shake the mixture. Let it sit in an ice bath for 5 min to facilitate precipitation of the proteins. Following this, centrifuge the samples at 15,000 *g*, 4 °C for 20 min. Once centrifuged, collect a certain amount of the supernatant and dilute it with mass spectrometry-grade water to obtain a methanol content of 53%. After dilution, centrifuge the samples again at 15,000 *g*, 4 °C for 20 min to remove any remaining impurities. Collect the supernatant and analyze it using liquid chromatography-mass spectrometry (LC-MS) for further analysis. The chromatographic conditions used for LC are as follows: Hypesil Goldcolumn (C18) is used as the chromatographic column, with the column temperature maintained at 40 °C, and a flow rate of 0.2 ml/min. For the positive mode, mobile phase A is 0.1% formic acid, and mobile phase B is methanol. Conversely, for the negative mode, mobile phase A is 5 mM ammonium acetate with a pH of 9.0, and mobile phase B is methanol. Regarding the mass spectrometry conditions, scans were performed between m/z 100 and 1500. An electrospray ionization (ESI) source was used with the following settings: spray voltage of 3.2 kV, sheath gas flow rate of 40 arb, aux gas flow rate of 10 arb, and a capillary temperature of 320 °C. The polarity can be set in either positive or negative mode. For tandem mass spectrometry (MS/MS), data-dependent scans are performed.

The raw data files (.raw) obtained from the spectrometry analysis were imported into the advanced software Compound Discoverer 3.1 (CD3.1, Thermo Fisher) for further analysis. Initial screening of the data was performed based on parameters such as retention time and mass-to-charge ratio. To improve identification accuracy, peak alignment was performed on different samples, using a retention time deviation of 0.2 min and a mass deviation of 5 ppm. Peak extraction was carried out based on the set parameters, which included a mass deviation of 5 ppm, signal intensity deviation of 30%, signal-to-noise ratio of 3, minimum signal intensity of 100,000 and summing ions. The obtained peak areas were then quantified, with target ions integrated accordingly. To improve identification accuracy further, molecular formula prediction was performed. This prediction relied on both molecular ion peaks and fragment ion information, and the results were cross-referenced with databases including mzCloud^1^, mzVault, and Masslist. To enhance data quality, blank samples were utilized for background ion removal. Subsequently, the quantification results underwent normalization, ultimately yielding data that included both identification and quantification outcomes. This analysis should provide you with a comprehensive and reliable overview of the analyzed substance (31).

### 2.16 Statistical analysis

GraphPad Prism software was employed for data analysis (version 8.0, United States). The value is expressed as Mean ± SEM. Statistical significance of group differences was assessed using one-way analysis of variance (ANOVA) followed by Tukey’s post-test. For data that did not follow a normal distribution, the Kruskal-Wallis one-way ANOVA was employed. Statistical differences are represented by *P*-values less than 0.05.

## 3 Results

### 3.1 Identification of components in GDQ by LC-MS/MS

**FIGURE 1:**
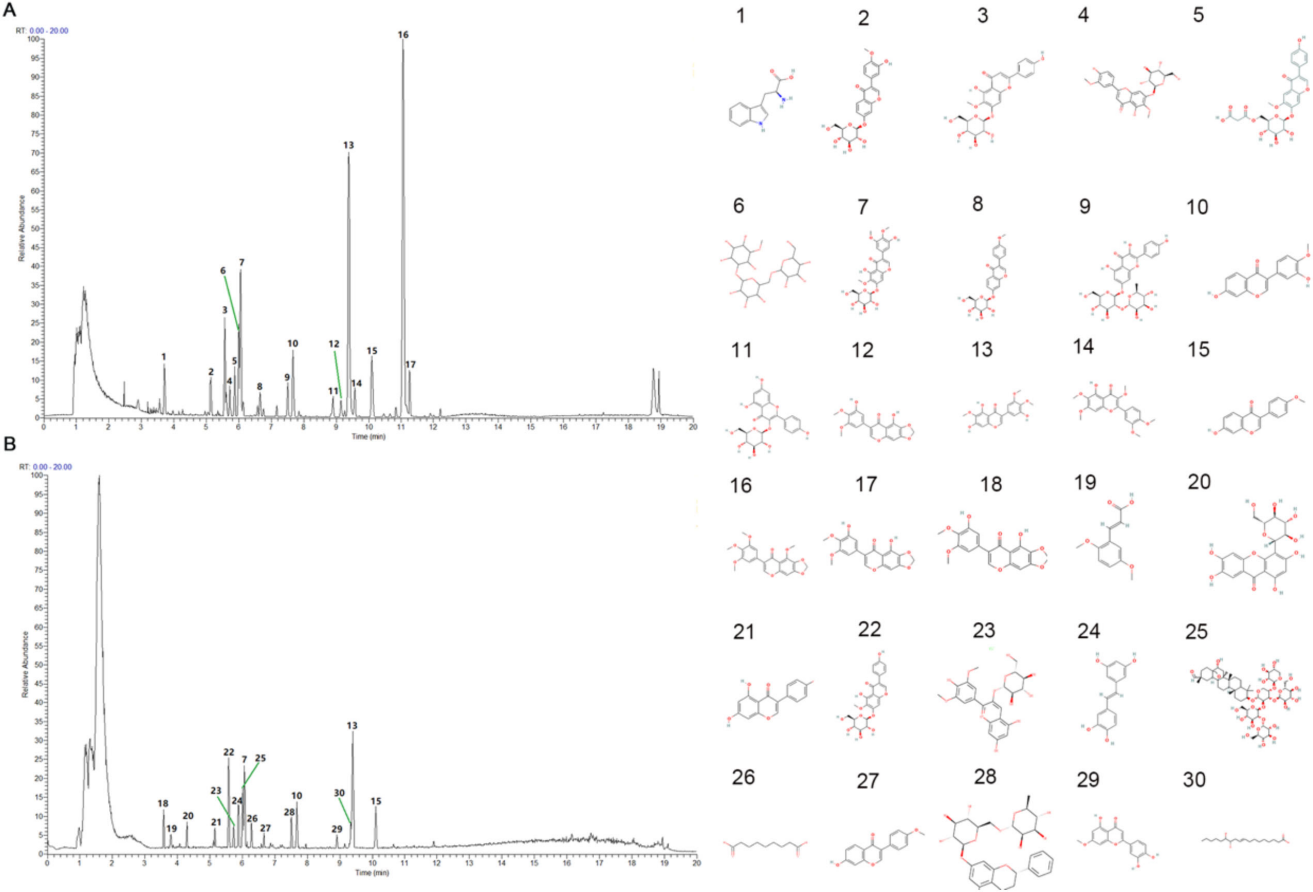
GDQ positive and negative BPC plot. **(A)** BPC plot in positive ion mode of GDQ - standard peak. **(B)** BPC plot of GDQ in GDQ negative ion mode — standard peak. 1–30 represents the molecular formula of each active ingredient.

In this experiment, UHPLC-HRMS was used to collect the data of GDQ solution and compare its positive and negative ion BPC maps. In the GDQ sample (GDQ positive and negative ion BPC diagram, the chromatographic peaks with higher abundance were confirmed for peak shape and examined for secondary spectrum, and then the peak numbers were labeled, and each peak in the positive and negative ion diagram was labeled in digital order as shown in the Figures 1A, B. We will include a detailed table as attachments to highlight key findings (Supplementary Material 1).

The collected data were imported into the local standard map database of traditional Chinese medicine for secondary mass spectrometry search and comparison, 1051 chemical components in GDQ solution were analyzed and identified, and a total of 99 categories were identified according to the classification method in the literature Classy Fire. Among them, the top 6 compounds and their subclasses are: Benzene and substituent derivatives, Carboxylic acids and derivatives, Fatty Acyls, Flavonoids, Organooxygen compounds, and Prenol lipids (Table 1).

**TABLE 1:** Category and quantity of compound in GDQ.

	Class1	Subclass	Freq	Class_ID	Subclass_ID
1	Benzene_and_substituted_derivatives	Anilides	7	Class_1(93)	Sub_2
		Benzoic_acids_and_derivatives	33		Sub_1
		Other	53		Other
2	Carboxylic_acids_and_derivatives	Amino_acids_peptides_and_ analogs	126	Class_2(144)	Sub_1
		Other	13		Other
		Tricarboxylic_acids_and_derivatives	5		Sub_2
3	Fatty_Acyls	Fatty_acids_and_conjugates	41	Class_3(101)	Sub_1
		Lineolic_acids_and_derivatives	24		Sub_2
		Other	36		Other
4	Flavonoids	Flavonoid_glycosides	56	Class_4(92)	Sub_1
		*O*_methylated_flavonoids	24		Sub_2
		Other	12		Other
5	Organooxygen_compounds	Carbohydrates_and_carbohydrate_conjugates	58	Class_5(94)	Sub_1
		Carbonyl_compounds	20		Sub_2
		Other	16		Other
6	Prenol_lipids	Other	43	Class_6(81)	Other
		Terpene_glycosides	23		Sub_1
		Triterpenoids	15		Sub_2

### 3.2 Real-ambient PM_2.5_ exposure

**FIGURE 2:**
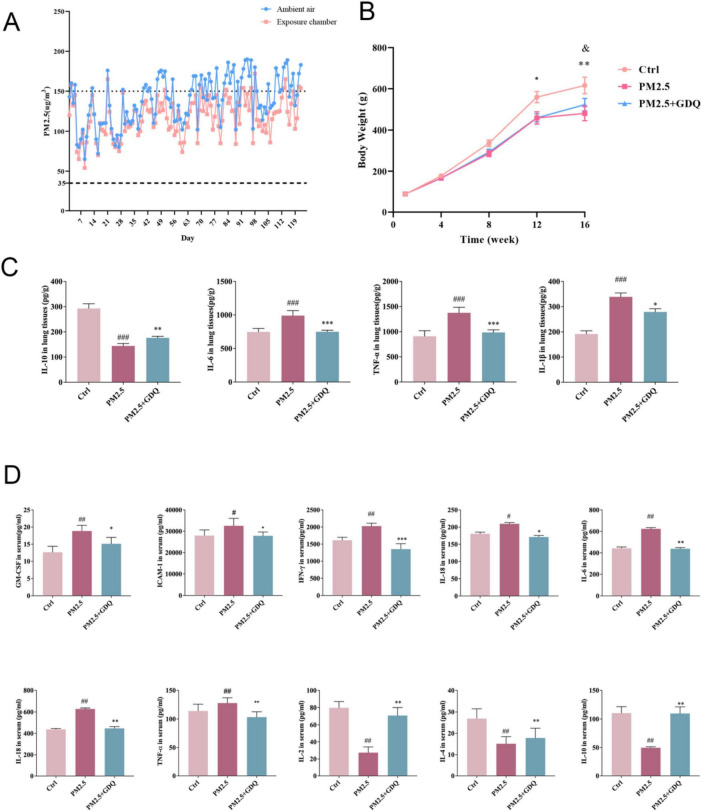
GDQ inhibit systemic inflammatory response induced by PM_2.5_ exposure. **(A)** Concentrations of PM_2.5_ in PM_2.5_ exposure chambers and ambient air recorded during the experiment. Dashed and dotted lines represent the average daily limits and levels of severe air pollution for the air quality guidelines in China. **(B)** Body weights of rats were recorded during the experiment (*n* = 20). **(C)** The expression of IL-10, IL-6, TNF-α, and IL-1β were determined by ELISA. **(D)** Expression level of inflammatory factors in BALF. Data are presented as mean ± SEM. #*p* < 0.05, ##*p* < 0.01, ###*p* < 0.001 compared to control group. *p < 0.05, **p < 0.01, ***p < 0.01, compared to PM_2.5_ group.

Throughout this experiment, rats were continuously exposed to PM_2.5_ for 16 weeks, 24 h a day, 7 days a week. The detailed experimental procedure is illustrated in Supplementary Figures 1A, B. During the exposure period in Jinniu District, Chengdu, the mean PM_2.5_ concentration in the surrounding environment was 117.8 μg/m^3^, while the PM_2.5_ concentrations in the dedicated PM_2.5_ exposure chambers were recorded as 110.5 μg /m^3^ (Figure 2A). Statistical analysis revealed that the PM_2.5_ concentrations in the exposure chambers were comparable to those in the ambient environment. Following filtration through the filters, the PM_2.5_ concentration in the AF room decreased significantly, indicating the filters’ effectiveness in acting as a barrier against PM_2.5_. Previous studies have also investigated the composition of PM_2.5_ in indoor settings, which closely resemble the components found in the ambient environment.

### 3.3 GDQ inhibit systemic inflammatory response induced by PM_2.5_ exposure

During the entire study, we observed that the rats exposed to PM_2.5_ inhaled air showed a slower increase in body weight compared to the rats in the AF chamber (Figure 2B). However, this trend was effectively reversed following the intervention of GDQ. Additionally, following GDQ intervention, the rats displayed significantly shinier fur in comparison to the PM_2.5_ exposed indoor rats, indicating an improvement in their overall health. Furthermore, the activity levels of the rats were notably enhanced after GDQ intervention, illustrating a greater sensitivity and vitality.

Since the lungs are the main organ affected by PM_2.5_ exposure, our initial emphasis was on evaluating the inflammatory responses occurring in the respiratory tract. To investigate the effect of PM_2.5_ on lung tissue inflammation, we measured the expression levels of inflammatory cytokines, including IL-10, IL-6, IL-1β, and TNF-α, in lung tissues using ELISA (Figure 2C). At the same time, we also used microarray inflammatory factor kit to detect the expression level of inflammatory factors in BALF (Figure 2D). The results revealed significantly elevated cytokine levels in the PM_2.5_ exposed group, in comparison to the AF group. Moreover, GDQ intervention successfully reduced cytokine levels when compared to the PM_2.5_ group.

### 3.4 GDQ regulated systemic and local immune response of PM_2.5_ induced lung injury

**FIGURE 3:**
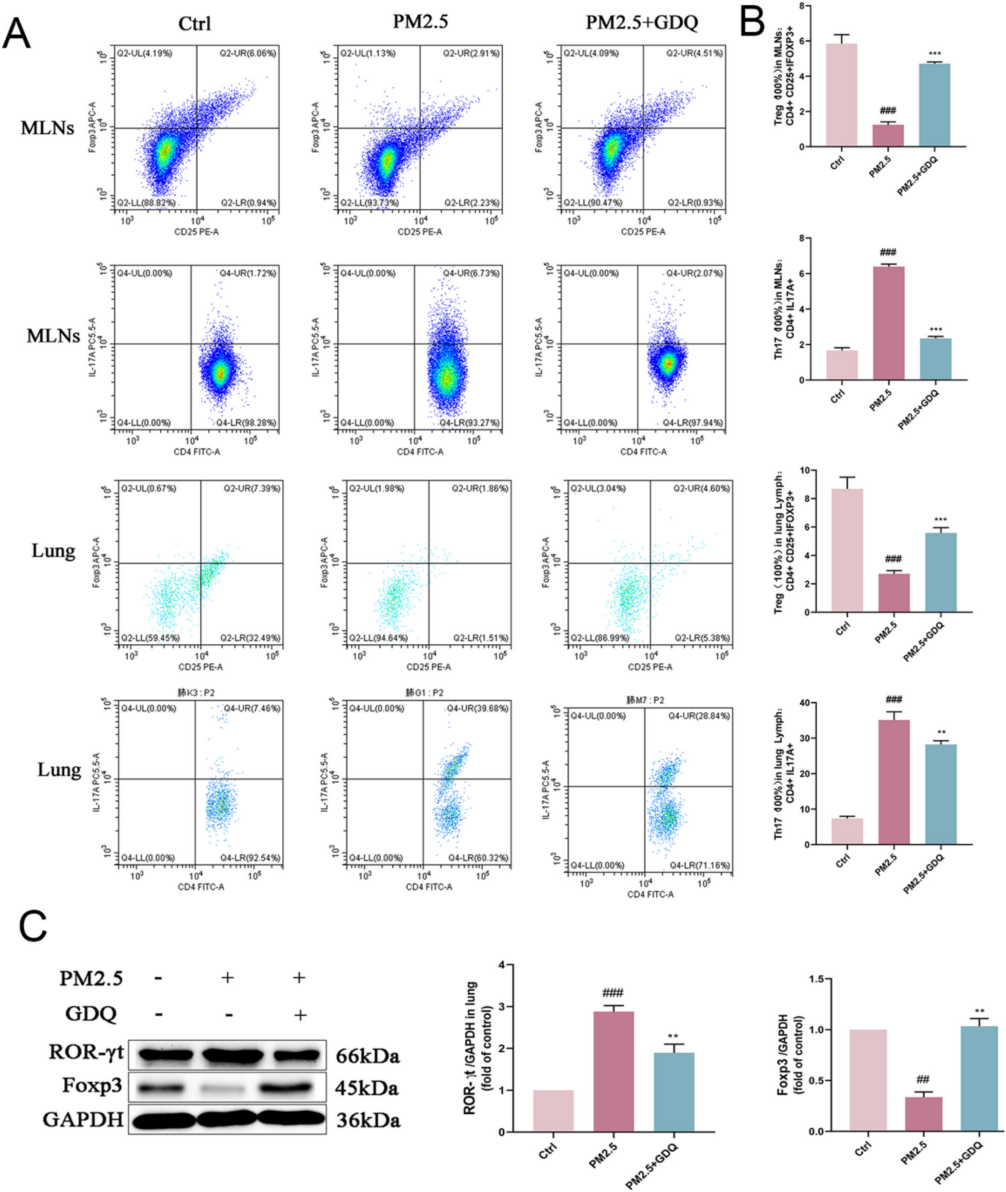
GDQ rescued Th17/Treg imbalance in PM_2.5_-induced lung injury. **(A)** Representative FACS plots of IL-17A^+^ cells in the CD4^+^ T-cell subset and Foxp3^+^ cells in CD4^+^CD25^+^ T-cell subset in the lung tissues and MLNs. **(B)** Statistical chart of expression of Th17 and Treg cells. **(C)** The expression of Foxp3 and ROR-γt protein were determined by Weston blot. Data are presented as mean ± SEM (*n* = 7). ##*p* < 0.01, ###*p* < 0.001 compared to control group. **p < 0.01, ***p < 0.001 compared to PM_2.5_ group.

The lung’s immune system was partly propelled by the growth and diversification of immune cells within the lung and MLNs. Investigating GDQ’ impact on immune cells within lung tissues and MLNs, the specific subset of T cells in these areas was identified using flow cytometry. We selected Foxp3 as a marker for regulatory T cells (Tregs) and ROR-γt as a marker for Th17 cells due to their well-established roles in immune regulation and inflammation, particularly in the context of lung injury and microbiota interactions (32, 33). Tregs, characterized by Foxp3 expression, are crucial for maintaining immune tolerance and suppressing excessive inflammatory responses, which are key factors in mitigating lung injury. On the other hand, ROR-γt is a hallmark of Th17 cells, which are involved in promoting inflammatory responses and are often implicated in immune dysregulation associated with lung diseases. The results showed a decrease in Treg count in CD4^+^ T cells and an increase in Th17 cells in the PM_2.5_ group relative to rats in the AF group. GDQ escalated the proliferation of Treg cells within lung tissues and MLNs, whereas their reduction in Th17 cell expression mirrored that observed in AF rats (Figure 3A). At the same time, we performed statistical analysis of the data to more intuitively see the expression levels of Th17 cells and Treg cells (Figure 3B). Consequently, we simultaneously analyzed how ROR-γt and Foxp3 are expressed in lung tissues (Figure 3C). Relative to the AF group, there was a notable surge in ROR-γt expression in the PM_2.5_ group, whereas Foxp3 expression in the PM_2.5_ group exhibited a slight decrease relative to the AF group. Alterations in the previously mentioned variables can be reversed following GDQ therapy.

### 3.5 GDQ ameliorate pulmonary dysfunction caused by PM_2.5_ exposure

**FIGURE 4:**
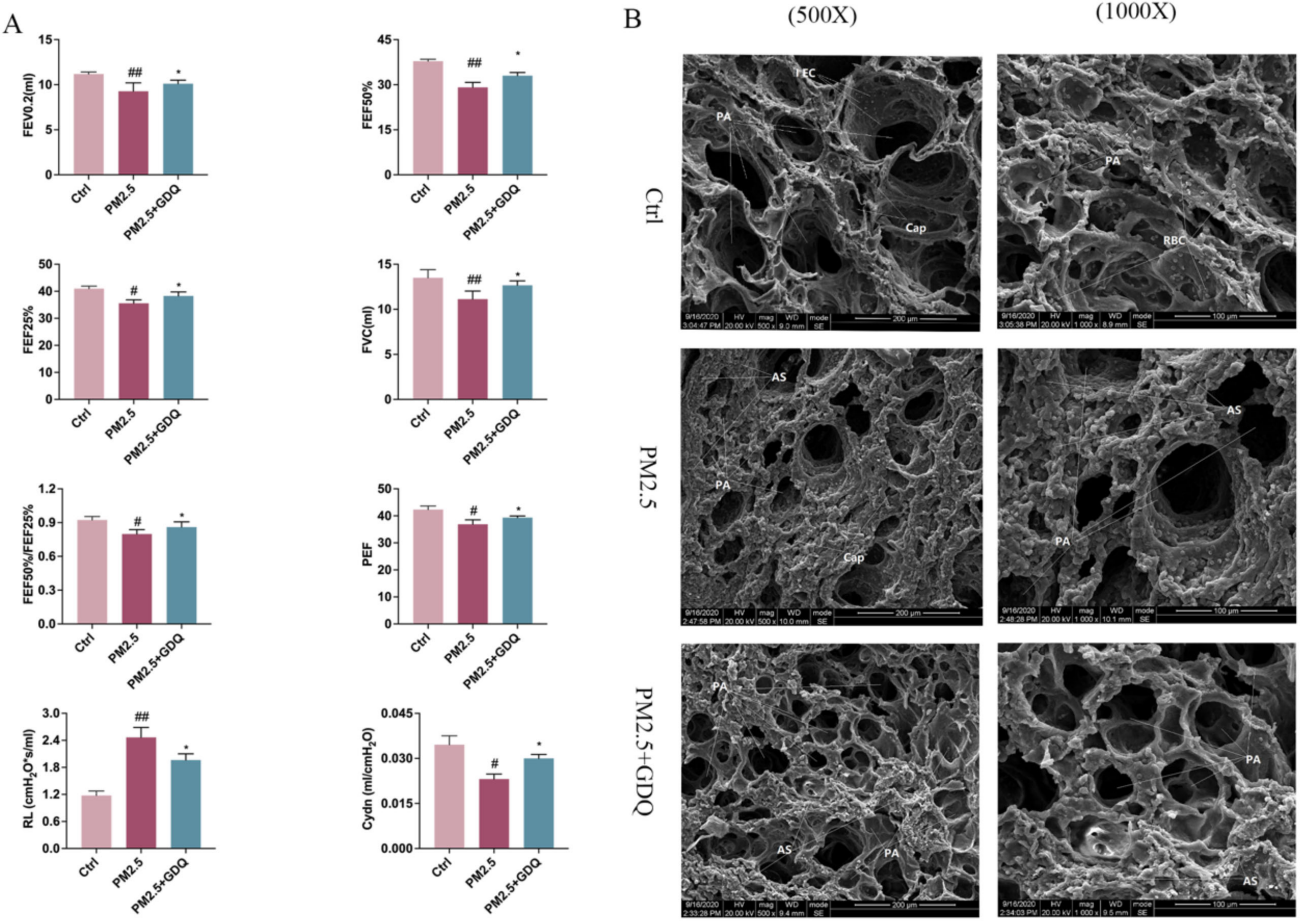
GDQ protected against PM_2.5_-induced lung injury. **(A)** Pulmonary function instruments were used to detect pulmonary function parameters under anesthesia in rats (*n* = 7), Lung function parameters: FVC, forced vital capacity; FEV0.2, forced expiratory volume in the first 0.2 s; FEF25%, maximal flow at 25% of forced vital capacity; FEF50%, maximal flow at 50% of forced vital capacity; PEF, peak expiratory flow; RL, resistance of lung; Cydn, Respiratory dynamic compliance. **(B)** PM_2.5_ induces lung damage to the ultrastructure of alveolar macrophages in lung tissue. (The first row of panel **(B)**: scale bar = 1 μm, SEM × 500, The second row of panel **(B)**: scale bar = 2 μm, SEM × 1000). Data are presented as mean ± SEM. #*p* < 0.05, ##*p* < 0.01, compared to control group. *p < 0.05, compared to PM_2.5_ group.

To further investigate the detrimental impacts of PM_2.5_ exposure on lung health, we conducted lung function measurements in rats. Currently, invasive lung function tests represent the most reliable method for evaluating lung function in rats, serving as the gold standard for such assessments (34). In our study, lung function was assessed using the FOT. The PM_2.5_-exposed rats exhibited significantly reduced Forced Vital Capacity (FVC) in comparison to the AF group, accompanied by reductions in the 0.2 s forced expiratory volume (FEV0.2), as well as reductions in the ratio of 50% FVC to 25% FVC (F50/F25%) in the PM_2.5_ exposed rats (Figure 4A). Peak expiratory flow (PEF) is a vital measure, as it reflects the highest rate of exhalation through the bronchial tree during forced expiration. PEF is frequently employed to assess the presence of airway obstruction. PEF serves as an indicator of bronchial hyperresponsiveness and mirrors alterations in airway caliber (35). PEF exhibited a statistically significant increase in PM_2.5_-exposed rats when compared to AF control rats. However, GDQ intervention could increase lung function parameters compared with control group. These results further illustrate that GDQ intervention reversed PM_2.5_ exposure-induced lung function decline in rats. At the same time, we detected dynamic lung compliance (Cydn) and lung resistance (RL) to assess the condition of pulmonary airway obstruction, and the study showed that GDQ intervention could improve pulmonary airway obstruction.

Scanning electron microscopy was employed to examine the ultrastructural changes in lung tissue. In the Ctrl group, lung tissue exhibited a pristine structure, with evenly distributed round-shaped alveoli. The type I epithelial cells of the alveolar wall were flat and firmly attached, displaying a wide coverage area. The cell membranes were intact, and no visible damage was observed. The alveolar septa were thin and uniform in thickness, with scattered red blood cells in the capillary lumens. In contrast, in the PM_2.5_ group, lung tissue sections exhibited more pronounced structural damage. Alveoli varied in size, with significant atrophy and irregular collapse. The type I epithelial cells of the alveolar wall were flat and attached to hypertrophied pulmonary interstitium. Large areas of the alveolar septa were noticeably thickened, with scattered red blood cells in the capillary lumens. In the GDQ group, the structural damage to lung tissue sections was mild. Alveoli were evenly distributed, with only a few varying in size. While individual alveoli showed slight atrophy, most of them maintained a round shape. The type I epithelial cells in the alveolar wall were flat, adherent, and displayed a wide coverage area. The cell membranes appeared intact, and no observable damage was detected. Mild pulmonary interstitial hypertrophy was observed, along with localized thickening of the alveolar septa. Notably, the pulmonary interstitium was mildly hypertrophic, while focal areas of the alveolar septa displayed thickening (Figure 4B).

### 3.6 GDQ alleviates PM_2.5_-induced lung tissue damage

**FIGURE 5:**
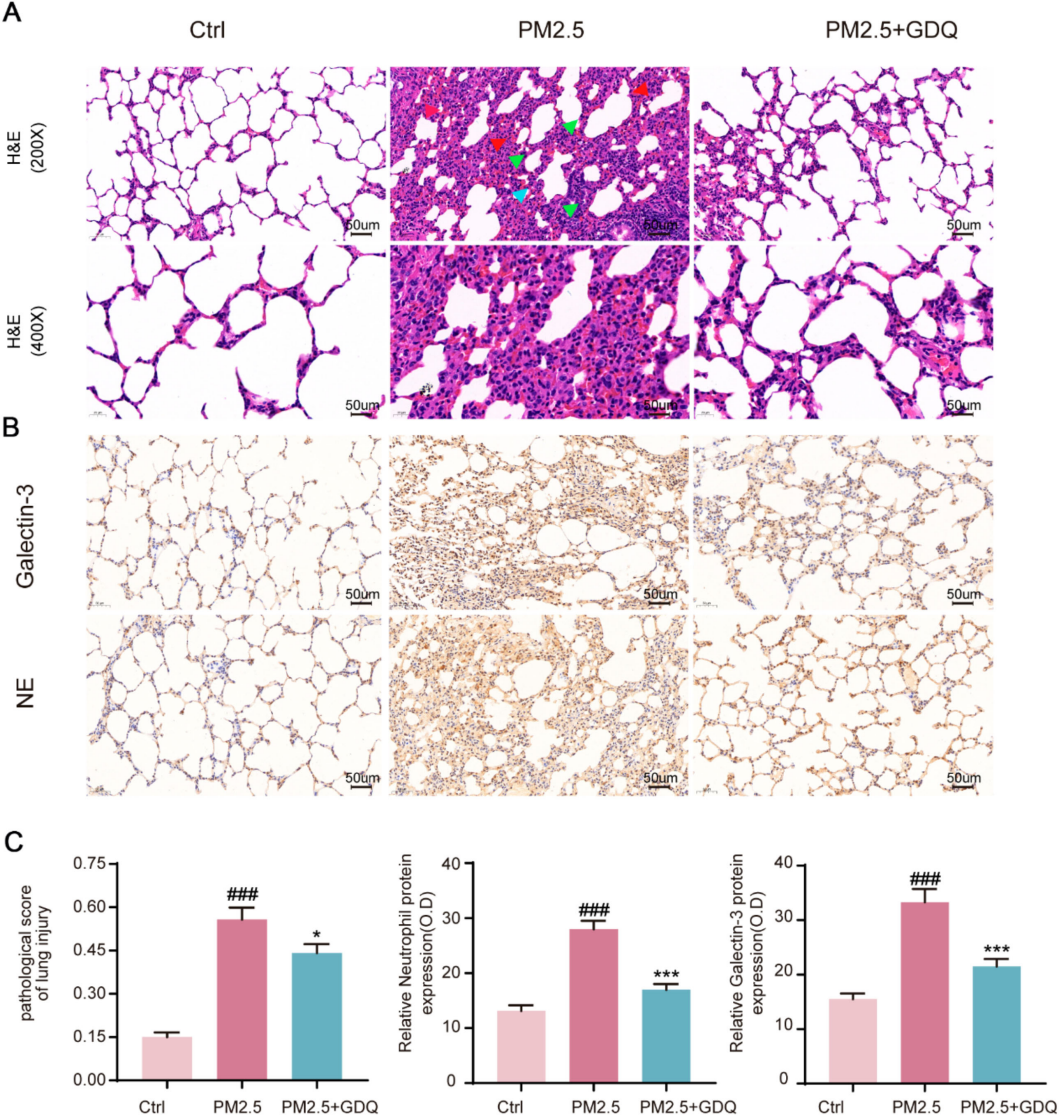
GDQ attenuates lung tissue damage induced by PM_2.5_. **(A)** HE staining of lung tissue in each group. (The first row of Figure 3A: magnification 200 ×, Scale bar = 50 μm, The second row of Figure 3A: magnification 400 ×, Scale bar = 50 μm). Arrows indicate classical lung injury. The blue, red and green arrows indicate inflammatory cell infiltration, hyperaemia, and thickening of the alveolar walls, respectively. **(B)** Immunohistochemical staining (magnification × 200) was used to observe the effect of GDQ on neutrophil and galectin-3 expression in lung tissue of PM_2.5_ induced lung injury in rats. **(C)** Statistical plots of semiquantitative histological scores and positive expression of neutrophils and galectin-3 assessed for lung injury induced by PM_2.5_. Data are presented as mean ± SEM (*n* = 7). ###*p* < 0.001, compared to control group. * *p* < 0.05, *** *p* < 0.001compared to PM_2.5_ group.

To further examine the histological changes in lung tissues, we conducted a pathological analysis. The obtained lung histopathology data revealed that exposure to PM_2.5_ induced severe bronchitis and interstitial pneumonia, characterized by thickening of the alveolar wall, congestion in the alveolar space, and significant pulmonary edema. However, following GDQ intervention, both bronchitis and pulmonary inflammatory cell infiltration were mitigated. These results indicate a clear association between PM_2.5_ exposure and lung injury, as evidenced by the decline in lung function, while GDQ intervention proved effective in alleviating this injury (Figure 5A).

Immunohistochemical staining with anti-neutrophils and galectin-3 (a macrophage specific marker) demonstrated considerable infiltration of neutrophils and macrophages in the lungs of rats exposed to PM_2.5_ (Figure 5B). Notably, the lung tissue of rats in the PM_2.5_ exposed group exhibited severe damage, with a marked increase in macrophage and neutrophil infiltration when compared to the AF group (Figure 5C). However, GDQ intervention significantly alleviated inflammatory cell infiltration induced by PM_2.5_ exposure. Collectively, these findings provide compelling evidence that GDQ effectively mitigates inflammatory responses in PM_2.5_ exposed rats.

### 3.7 GDQ’s effect on lung microbiota composition induced by PM_2.5_ exposure in rats

**FIGURE 6:**
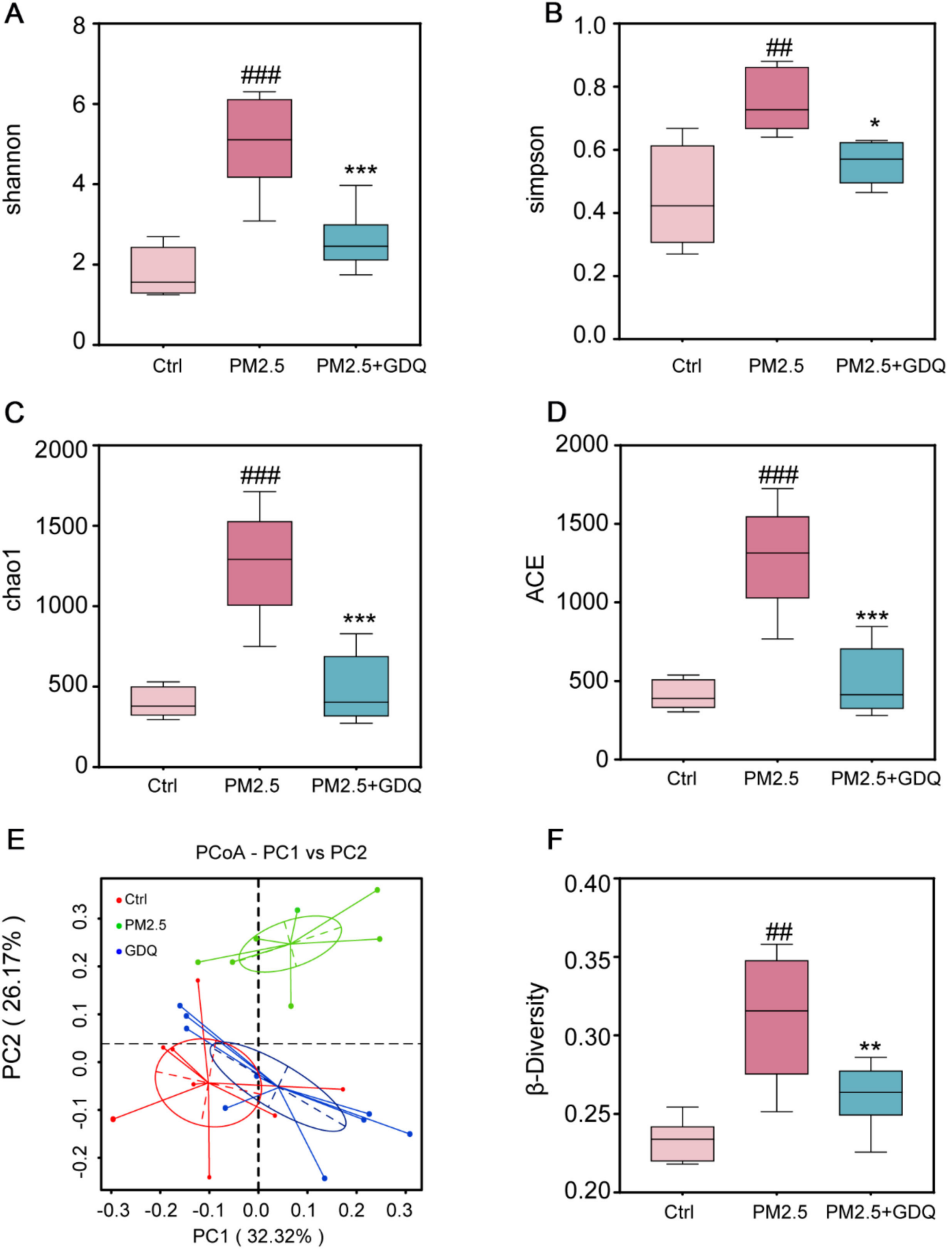
The effect of GDQ on the composition of the lung microbiota induced by PM_2.5_ exposure in rats. **(A)** Shannon index, **(B)** Simpson index, **(C)** chao1 index, **(D)** ACE index, **(E)** weighted UniFrac principal coordinate analysis (PCoA), **(F)** β-diversity of lung microbiota in each group (*n* = 6). ##*p* < 0.01, ###*p* < 0.001, compared to control group. * *p* < 0.05, ** *p* < 0.01, *** *p* < 0.001, compared to PM_2.5_ group.

We utilized 16S rRNA gene sequencing to explore how PM_2.5_ exposure affected the lung microbiome and to assess the potential effects of GDQ intervention. Alpha diversity metrics were utilized to gauge the diversity and richness of the lung microbial communities. The results demonstrated an increase in microbial community diversity in rats exposed to PM_2.5_ compared to the Ctrl group, as indicated by both the Shannon index and Simpson index (Figures 6A, B). However, GDQ intervention led to a reduction in lung microbiome diversity. Additionally, the richness estimates of Chao1 and ACE were substantially higher in the PM_2.5_ group than in the Ctrl group, and GDQ intervention resulted in decreased values of Chao1 and ACE (Figures 6C, D).

Weighted UniFrac principal component analysis (PCoA) was utilized to evaluate the similarity of microbiota and detect variations in lung microbiota composition among the groups. The PCoA results demonstrated dissimilarities in the composition and structure of lung microbiota between the PM_2.5_ group and the other groups. This finding suggests that the stimulation of PM_2.5_ significantly perturbed the delicate equilibrium of lung microbiota, disrupting its dynamic balance (Figure 6E). Significantly, it was observed that the GDQ and Ctrl groups exhibited a closer distance, suggesting that GDQ administration partially alleviated the lung dysbiosis triggered by PM_2.5_ exposure. We used the beta diversity index in this research to assess potential variations in species diversity among the groups, applying the Wilcoxon rank-sum test for analysis (Figure 6F). After analyzing the box plots presented in the figure, it can be observed that the width of the box within each group suggests good reproducibility. Moreover, the PM_2.5_ group displayed a rise in microbiota diversity in contrast to the control group. However, following GDQ intervention, the diversity decreased, and this difference was statistically significant.

**FIGURE 7:**
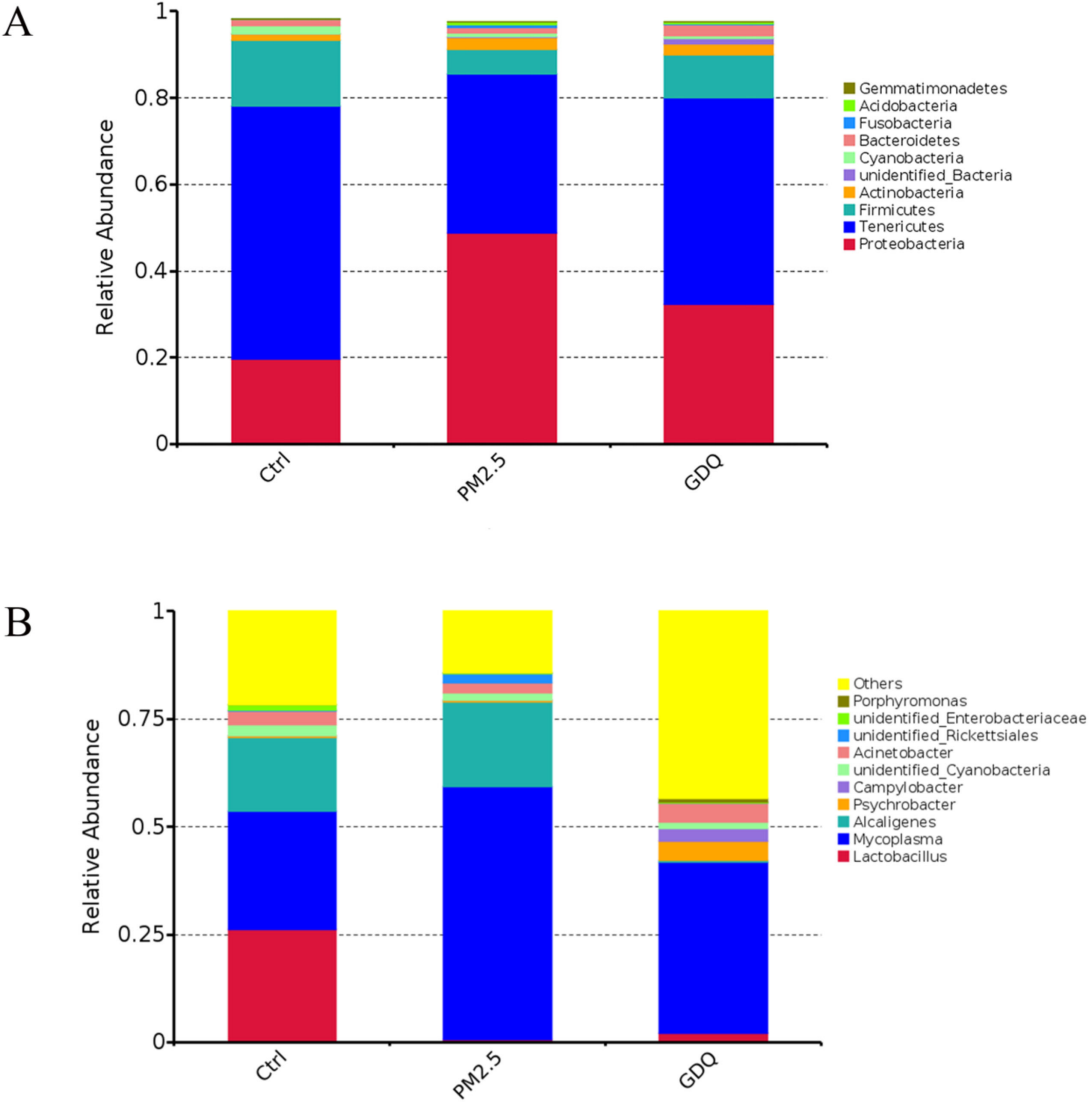
The effect of GDQ on the composition of the lung microbiota induced by PM_2.5_ exposure in rats. The lung microbiota composition profiles at the phylum **(A)** and genus **(B)** level.

To further investigate the variation in species, we examined the lung bacterial composition and their relative abundance. The taxonomic composition was assessed at both the phylum and genus levels to identify significant differences among the groups. The taxonomic composition at the phylum level mainly comprises: *Proteobacteria*, *Tenericutes*, *Firmicutes*, *Actinobacteria*, Notably, the administration of GDQ could ameliorate the alterations in lung microflora induced by PM_2.5_ exposure (Figure 7A). At the genus level, *Pseudomonas*, *Mycoplasma*, and *Lactobacillus* were the main dominant genera. *Lactobacillus* was higher than PM_2.5_ group after GDQ intervention, while *Pseudomonas* and *Mycoplasma* were lower than PM_2.5_ group. These findings suggest that GDQ intervention can modulate the bacterial structure in the lungs of PM_2.5_-exposed rats (Figure 7B).

### 3.8 Effects of GDQ on the serum metabolome in rats exposed to PM_2.5_

**FIGURE 8:**
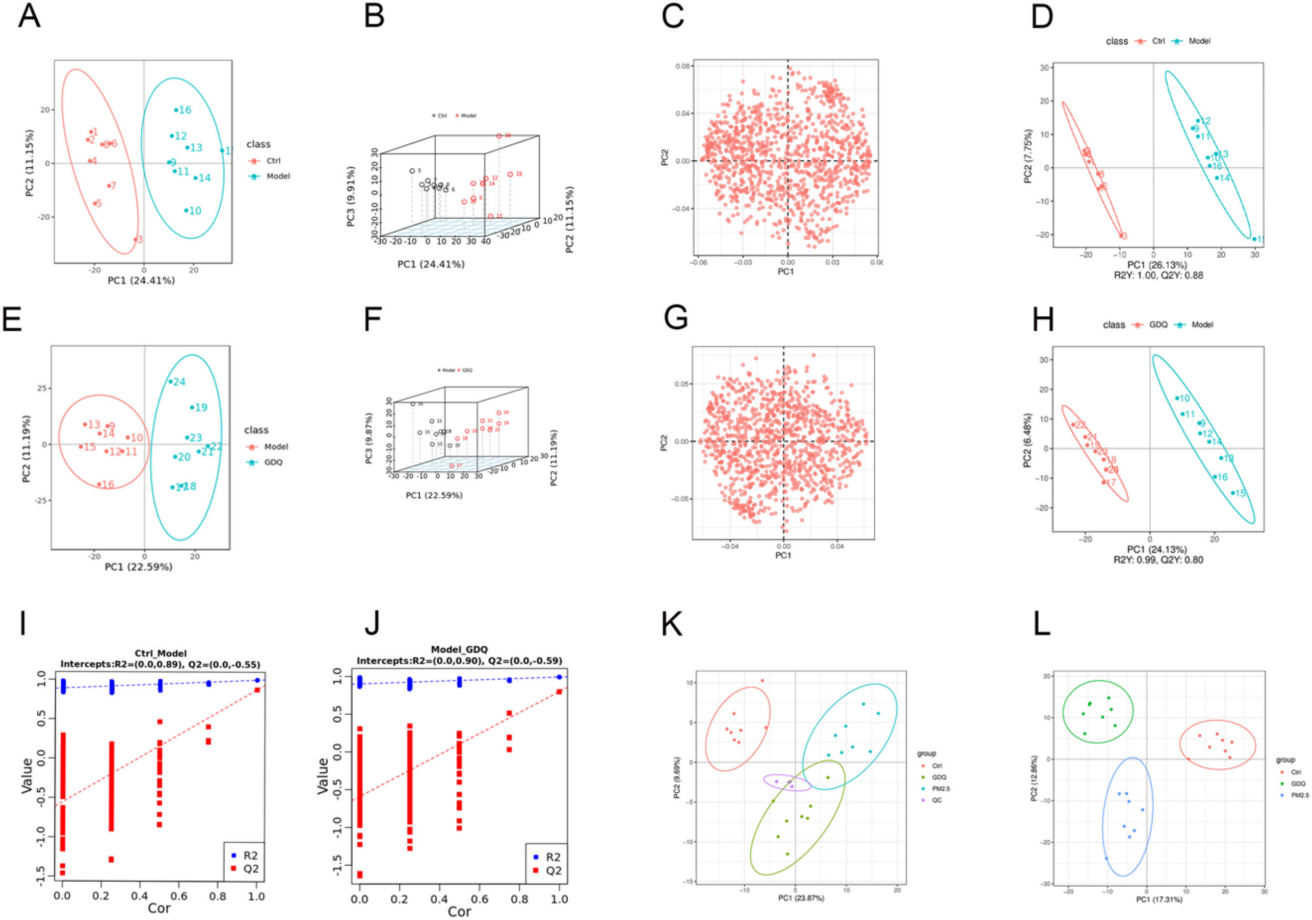
The effect of GDQ on the serum metabolome induced by PM_2.5_ exposure in rats. **(A)** PCA score plots in Ctrl and Model group. **(B)** 3D PCA plots in Ctrl and model group. **(C)** PCA loading plots in Ctrl and model group. **(D)** PLS-DA model validation diagram in Ctrl and model group. **(E)** PCA score plots in model and GDQ group. **(F)** 3D PCA plots in model and GDQ group. **(G)** PCA loading plots in model and GDQ group. **(H)** PLS-DA model validation diagram in model and GDQ group. **(I)** Validation plot of PLS-DA sorting in Ctrl and model group. **(J)** Validation plot of PLS-DA sorting in model and GDQ group. **(K)** Total PCA score plot in Ctrl and model group. **(L)** Total PCA score plot in model and GDQ group.

Analysis of the serum metabolome in rats from each group was conducted using LC-MS. The metabolites exhibited distinct separation between the Ctrl and PM_2.5_ groups, as evidenced by the principal components PC1 and PC2, which accounted for 24.41 and 11.15% of the total variation, respectively (Figure 8A). To gain a clearer understanding of the data, we created 3D PCA plots, which allow us to more easily interpret the data’s distribution and underlying structure (Figure 8B). We also produced PCA (principal component analysis) loadings plots to help understand the magnitude of each original variable ‘s contribution on principal components (Figure 8C). The PLS-DA scores revealed distinct dispersion between the control and PM_2.5_ groups, indicating significant differences in their metabolic profiles (Figure 8D). However, following GDQ intervention, the principal components PC1 and PC2 accounted for 22.59% and 11.19% of the total variation, respectively (Figure 8E). Similarly, we generated a 3D PCA plot to gain a clearer understanding of the data distribution (Figure 8F). PCA (principal component analysis) loadings plots to help understand the magnitude of each original variable ’s contribution on principal components (Figure 8G). The PLS-DA scores revealed distinct dispersion between the GDQ and PM_2.5_ groups, indicating significant differences in their metabolic profiles (Figure 8H). The validation results demonstrated the model’s robust predictive ability, thus providing additional evidence for the experiment’s reliability (Figures 8I, J). The drug intervention led to notable changes in metabolite characteristics, indicating that the drug is modulating metabolic pathways. Furthermore, this modulation is significantly distinct from the disease state. Notably, the metabolic profiles of the GDQ group exhibited closer proximity to those of the blank group, suggesting that GDQ intervention could ameliorate the metabolic disruptions caused by PM_2.5_ exposure (Figures 8K, L).

In this study, we employed variable importance projection (VIP) as a threshold to discern metabolites that exhibited notable variances between the Ctrl and PM_2.5_ groups. Metabolites that met the criteria of VIP > 1.0 and fold change (FC) > 1.5 were considered as differential metabolites. A total of 290 metabolites displayed significant differences between the Ctrl and PM_2.5_ groups in the comparative analysis.

**FIGURE 9:**
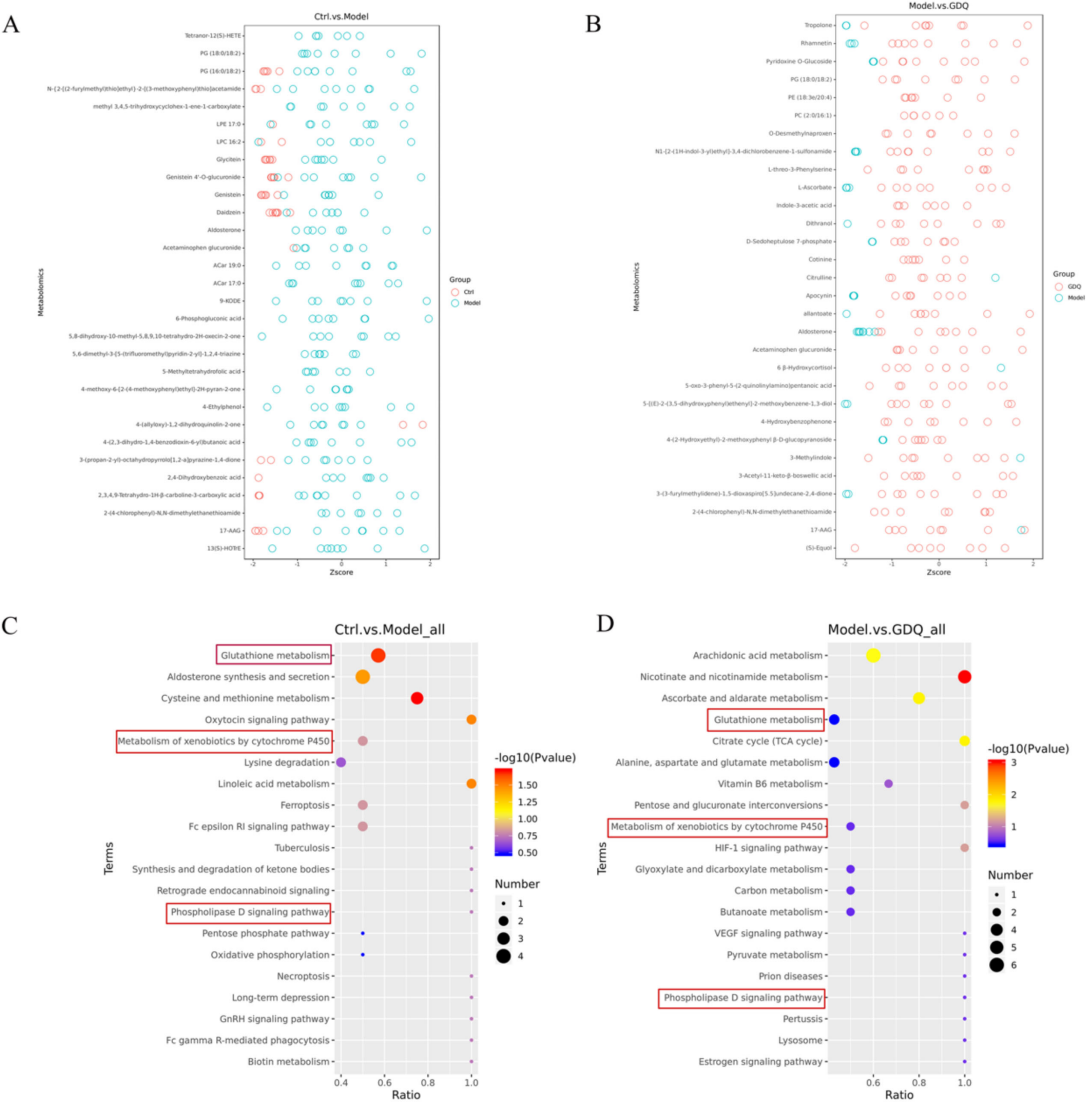
The effect of GDQ on the serum metabolome induced by PM_2.5_ exposure in rats. **(A)** Z-score plot of differential metabolite between Ctrl and PM_2.5_ group, **(B)** Z-score plot of differential metabolite between PM_2.5_ and GDQ group, **(C)** KEGG enriched bubble map between Ctrl and PM_2.5_ group, **(D)** KEGG enriched bubble map between PM_2.5_ and GDQ group.

Out of the 290 differential metabolites, 146 metabolites showed an elevation in the PM_2.5_ group, while 144 metabolites exhibited a decrease compared to the Ctrl group. We included a detailed table as attachments to highlight key findings (Supplementary Material 2–3). To gain further insights into the differences in metabolite profiles, a qualitative and quantitative analysis was conducted on the major metabolites identified in each group. The relative content of metabolites at the same level was assessed using Z-Score, and the top 30 metabolites were identified (Figure 9A). When comparing the PM_2.5_ group with the GDQ group, a total of 312 distinctive metabolites were identified. Among these 312 metabolic species, 206 metabolites exhibited an elevation, while 106 metabolites showed a decrease in the GDQ group compared to the PM_2.5_ group. Additionally, a qualitative and quantitative analysis was performed on the major metabolites identified in each group. The relative content of metabolites at the same level was assessed using Z-Score, and the top 30 metabolites were identified (Figure 9B). The different metabolites were analysed using the KEGG database, and metabolic pathways were constructed and analysed to gain insight into the relevant metabolic pathways associated with the inflammatory response. The analysis revealed that these metabolites are involved in various metabolic pathways, including the Phospholipase D signaling pathway, Metabolism of xenobiotics by cytochrome P450, and Glutathione metabolism (Figures 9C, D). These findings shed light on the potential mechanisms underlying the action of the inflammatory response and provide valuable information for further investigation.

## 4 Discussion

With the rapid development of Chinese cities, the concentration of PM_2.5_ in the atmosphere has been increasing year by year, which in turn induces and aggravates the occurrence and development of respiratory diseases, such as COPD (36), asthma (37), and lung cancer (38). The adverse effects of PM_2.5_ on human and possible underlying mechanisms have been documented, however, the prevention and treatment methods are scarce. Therefore, it is necessary to explore preventive measures that can protect people from lung injury caused by PM_2.5_. In this study, we investigated the metabolic and pulmonary microbial changes in rats after systemic environmental PM_2.5_ exposure using untargeted metabolomics and 16S RNA sequencing in a real environmental exposure system, and the safeguarding impact of GDQ on PM_2.5_-triggered lung injury.

GDQ, a widely used traditional Chinese medicine formula, has shown certain efficacy in treating chronic airway inflammation; however, its precise mechanisms of action remain unclear. In this study, we intervened in a PM2.5-induced chronic airway inflammation model and demonstrated that GDQ alleviates pathological lung tissue damage and significantly improves the inflammation response induced by PM2.5. Through HE staining, immunohistochemical staining, and scanning electron microscopy, we observed that GDQ effectively reduced pathological changes in lung tissue, including decreased infiltration of inflammatory cells and damage to the lung airway structure. These findings are consistent with previous studies, suggesting that GDQ may alleviate PM2.5-induced chronic airway inflammation by modulating immune cell activity and promoting repair of lung tissue. Furthermore, we observed that GDQ treatment improved lung function, as indicated by enhanced respiratory parameters in the GDQ treated group compared to the PM2.5-exposed control group. This suggests that GDQ not only alleviates the inflammatory process but also contributes to the functional recovery of the lungs, which is crucial in the context of chronic airway diseases (39).

In this study, we chose Foxp3 and ROR-γt as immune cell markers because they specifically represent regulatory T cells (Tregs) and TH17 cells, respectively, which play key roles in immune balance during lung inflammation. Tregs (marked by Foxp3) suppress excessive inflammation, while TH17 cells (marked by ROR-γt) promote it. The balance between these cells is crucial in PM_2.5_-induced lung injury (40). By focusing on these markers, we aimed to explore how GDQ modulates this important immune axis. Other markers could provide insights, but these are most relevant to our study’s focus on immune regulation. Additionally, through flow cytometry, we analysed the balance between TH17 and Treg cells. TH17 cells, which promote inflammation, were significantly elevated in the PM2.5-exposed group, while Treg cells, which help maintain immune tolerance, were reduced (41). GDQ treatment restored the balance between these cell populations, lowering the proportion of TH17 cells and enhancing Treg cell activity. This indicates that GDQ may exert its anti-inflammatory effects by modulating immune responses, specifically by restoring immune homeostasis through the regulation of the TH17/Treg axis. According to the above results, we concluded that GDQ could effectively reduce lung injury induced by PM_2.5_.

Next, we analysed the effect of PM_2.5_ exposure on lung microbiome composition using 16S rRNA gene sequencing and observed the regulatory effect of GDQ on lung microbiota. The results showed that lung microbiota diversity and richness were higher in the PM_2.5_ group than in the control group, which was also consistent with previous studies. However, GDQ intervention could reduce the increase in the abundance and diversity of lung microbiota caused by PM_2.5_. In addition, our results showed that the main differential species were *Proteobacteria*, *Bacteroidetes*, *Cyanobacteria*, *Firmicutes*. The imbalance of these lung microbiome is present in a variety of respiratory diseases, and the increase of *Proteobacteria* is predominant, which is the main phylum leading to acute and COPD, pulmonary fibrosis and pulmonary sarcoidosis (42, 43). These results demonstrated that PM_2.5_ exposure disturbed the microbial structure of the lungs, and the microbial composition of the GDQ group was closer to that of Ctrl group, indicating that GDQ could restore the microbiota of the lungs. In fact, several studies have already shown that PM_2.5_ exposure can lead to lung injury, while lung microbes and airway immune system are intermediate mediators that increase individual sensitivity to PM_2.5_ (27, 28). Moreover, studies had also shown that lung microbes could interact with the airway immune system, indicating that PM_2.5_ exposure causing lung microbiome disorders might be one of the causes of lung injury (44). GDQ may enhance the activity and proliferation of Tregs, which can help restore immune balance disrupted by PM_2.5_ exposure. This immune regulation can create a more favorable environment for beneficial microbiota to thrive, thereby contributing to the restoration of lung microbial communities. PM_2.5_ exposure is known to promote Th17 cell differentiation, leading to increased inflammation and dysbiosis. GDQ’s ability to reduce ROR-γt expression and inhibit Th17 cell activity can mitigate the inflammatory response, thereby reducing the hostile environment that disrupts microbial communities. The lung microbiota and immune system are in constant interaction, with microbial metabolites and components influencing immune cell function and vice versa. By modulating Treg and Th17 cell populations, GDQ may alter the local cytokine milieu and other immune signals, indirectly promoting the growth of beneficial microbial species and suppressing pathogenic ones. This bidirectional interaction could be a key mechanism by which GDQ facilitates the restoration of a healthy lung microbiome. Therefore, our study demonstrates that PM2.5 exposure induces dysbiosis of the lung microbiota, which further exacerbates pulmonary inflammation and immune responses. GDQ is able to restore the structure of the lung microbiota, which may contribute to its anti-inflammatory effects. The balance of the microbiota is critical for maintaining immune system stability; thus, regulating the lung microbiota may be one of the key mechanisms by which GDQ alleviates PM2.5-induced lung injury.

Moreover, the LC-MS metabolomics analysis revealed notable alterations in the serum metabolic profile following PM_2.5_ exposure. Furthermore, KEGG enrichment analysis indicated that these metabolites were primarily associated with the Phospholipase D signaling pathway, Metabolism of xenobiotics by cytochrome P450, and Glutathione metabolism. These findings highlight the potential metabolic pathways involved in the response to PM_2.5_ exposure and the protective effects of GDQ intervention. Phospholipase D signaling pathway has been shown to play a key role in pulmonary vascular endothelial barrier dysfunction and can directly lead to lung injury (45, 46). The Metabolism of xenobiotics by cytochrome P450 signaling pathway is known to play a crucial role in the metabolism of polycyclic aromatic hydrocarbons and nitro compounds in the lung. Activation of this pathway can result in DNA damage and contribute to the development of lung tumors (47). Additionally, the glutathione metabolism pathway has been identified as a significant biomarker of pulmonary endoplasmic reticulum (ER) stress and is crucial in the pathogenesis of lung injury (48, 49). These changes in metabolic pathways suggest that GDQ may reduce lung oxidative stress and cellular damage caused by PM2.5 by regulating redox reactions and metabolism of harmful substances, thereby reducing inflammatory responses.

Studies have shown that beneficial shifts in the microbiome may generate metabolic by-products that influence the host’s metabolism (50). For example, *Lactobacillus* and *Bifidobacterium* are known to promote the biosynthesis of essential amino acids and lipids, which play important roles in cell repair and immune regulation (51). These microbial metabolites can impact key metabolic pathways, such as glutathione metabolism and phospholipase D signaling (52), which are crucial in the regulation of oxidative stress and inflammation. In our study, we found that GDQ treatment induces significant changes in the pulmonary microbiome, with beneficial bacteria such as *Lactobacillus* and *Bifidobacterium* increasing, which is associated with anti-inflammatory effects and improved lung health (53). These beneficial bacteria are involved in pathways related to lipid metabolism, amino acid synthesis, and energy production, contributing to the restoration of lung function.

The beneficial shift in the microbiome may also generate metabolic by-products that impact host metabolism. For example, *Lactobacillus* and *Bifidobacterium* are known to promote the biosynthesis of essential amino acids and lipids, which play important roles in cell repair and immune regulation (54). These microbial metabolites can influence key metabolic pathways, such as glutathione metabolism and phospholipase D signaling, which are critical in the regulation of oxidative stress and inflammation. Our metabolomics analysis identified significant changes in key metabolites, including a reduction in pro-inflammatory metabolites and an increase in anti-inflammatory and antioxidant metabolites, such as citrate, α-ketoglutarate, and fumarate. These metabolites are involved in pathways that reduce oxidative stress and enhance cell repair processes. The increase in beneficial microorganisms such as *Lactobacillus* and *Bifidobacterium* may support the production of these metabolites, thereby helping to alleviate lung damage and inflammation caused by PM2.5 exposure. Furthermore, microbial metabolites may modulate the host immune response through interactions with immune cells, particularly regulatory Tregs and TH17 cells (55). Tregs help maintain immune tolerance, while TH17 cells promote inflammation. GDQ treatment restored the balance between these cells, with reduced TH17 cell activity and enhanced Treg cell function. This immune modulation further explains how changes in the microbiome contribute to the improvement of lung injury. Importantly, changes in metabolic pathways, such as the regulation of glutathione metabolism, phospholipase D signaling, and cytochrome P450-mediated xenobiotic metabolism, are likely influenced by alterations in the microbiome composition. These pathways are involved in cellular detoxification, oxidative stress response, and inflammation regulation, which are crucial for alleviating lung injury. Shifts in the microbiome composition may generate metabolites that impact these pathways, thereby enhancing the recovery of lung function.

We have identified major compound peaks observed under both positive and negative ionization modes using a combination of mass spectrometry databases and literature search. The identified compounds include indoles and their derivatives, phenylpyrans, flavonoids, isoflavones, organoxins, stilbenes, lipids, and other compounds. At the same time, we analyzed the function of these compounds to investigate how the active ingredients in GDQ alleviate PM_2.5_-induced chronic airway inflammation. Such as Indole compounds modulate the aryl hydrocarbon receptor (AhR) pathway, crucial for protecting against PM_2.5_-induced oxidative stress and inflammation (56). Benzopyran compounds, including coumarins, exhibit antioxidant and anti-inflammatory effects by scavenging PM_2.5_-induced free radicals and inhibiting inflammatory pathways like NF-κB (57). Flavonoids protect lung cells from oxidative damage, reduce immune cell infiltration, and modulate lung microbiota, supporting lung health (58). Stilbenes, such as resveratrol, reduce oxidative stress, downregulate pro-inflammatory cytokines, and protect the alveolar-capillary barrier (59). In our study, we identified several key active components of GDQ that are particularly relevant to its anti-inflammatory effects. For instance, Astragaloside IV, Jaceosidin, and Irigenin have been shown to exhibit significant anti-inflammatory properties in previous studies. Astragaloside IV (27, 60), a primary active ingredient in Astragalus, has demonstrated strong anti-inflammatory effects by inhibiting pro-inflammatory cytokine production and modulating immune responses. Similarly, Jaceosidin (61), a flavonoid isolated from the herb Artemisia, has been reported to reduce inflammation by suppressing NF-κB signaling pathways. Irigenin (62), a compound found in *Belamcanda chinensis*, is also known for its anti-inflammatory activity through the regulation of inflammatory mediators such as TNF-α and IL-6.

Emerging evidence suggests that the gut and lung microbiomes can influence immune responses through a process known as the “gut-lung axis” (63, 64). Changes in the pulmonary microbiome composition may alter local immune cell functions, including the differentiation and activity of Th17 and Treg cells. The beneficial microbiome promoted by GDQ may help restore immune homeostasis by reducing the pro-inflammatory activity of Th17 cells and enhancing the regulatory function of Treg cells. This suggests that the modulation of the Th17/Treg balance by GDQ could be an indirect effect mediated by its impact on the pulmonary microbiome.

In addition to its potential indirect effects through the microbiome, GDQ may also exert direct effects on immune cells. Previous studies have indicated that certain bioactive compounds in GDQ, such as flavonoids and other active ingredients, can modulate immune responses by interacting directly with immune cells (65). These compounds may influence key signaling pathways involved in immune responses, such as the NF-κB and TGF-β pathways, affecting the differentiation of naïve T cells into Th17 or Treg cells (66). By modulating these pathways, GDQ may directly promote the differentiation of Treg cells and suppress the expansion of Th17 cells, leading to a more balanced immune response. Therefore, we propose that GDQ may regulate the Th17/Treg balance through these mechanisms. The interaction between the microbiome changes induced by GDQ and its direct effects on immune cells may synergistically restore immune balance, alleviate inflammation, and promote tissue repair.

Despite the promising findings of this study, it is important to acknowledge several limitations. Firstly, there is a potential for non-uniform distribution of PM_2.5_ in systemic exposures compared to tracheal instillation. However, previous research has demonstrated that both exposure methods result in comparable pulmonary toxicity in rats following exposure to refractory particulate matter (67). Secondly, due to the complex composition of PM_2.5_, it was not possible to identify the specific components responsible for the observed changes. Furthermore, using rats with a standardized rat microbiota could provide a more comprehensive model to investigate individual variations in lung microbiota. Since GDQ will eventually be applied in clinical settings, we acknowledge the inherent limitations of the animal model used in this study, particularly its ability to fully replicate human responses. While animal models are crucial for understanding disease mechanisms and testing potential therapies, they cannot completely mimic the complexity of human physiology. Differences between rats and humans in lung anatomy, immune response, and metabolism may influence the translation of these results to human clinical settings. Therefore, we plan to conduct clinical trials in future studies to validate the therapeutic efficacy and safety of GDQ in treating PM2.5-induced lung injury.

At the same time, our current study is not yet comprehensive, and further research is needed to refine our findings and to elucidate the precise mechanisms of action of GDQ. Although serum metabolomics provides valuable insights into systemic metabolic changes, it may not fully represent the local metabolic alterations in the lungs that contribute to PM2.5-induced lung injury. Therefore, future research should include tissue-specific metabolomics, such as the analysis of metabolites in lung tissue and BALF, to gain a more comprehensive understanding of the metabolic processes occurring in the lungs. This would allow for a more accurate assessment of how GDQ regulates local metabolic pathways and provide insights into its therapeutic potential in the context of lung-specific injury. This approach will allow us to compare and correlate systemic and localized metabolic changes, thereby providing a more comprehensive understanding of the mechanisms underlying PM_2.5_-induced lung injury and the therapeutic effects of GDQ. In future LC-HRMS (liquid chromatography-high resolution mass spectrometry) analyses, the metabolite pool will be pre-fractionated to separate polar and non-polar metabolites. This step will facilitate easier metabolite identification and matching. In future experiments, we can employ additional detection methods to analyze the gut microbiota, such as 16S rRNA sequencing, metagenomic sequencing, and immunofluorescence, to assess changes in the microbial community composition. Finally, our experiment lacked a group in which GDQ administration and PM_2.5_ exposure were initiated simultaneously. In future studies, we will incorporate experimental groups where GDQ treatment is administered concurrently with PM_2.5_ exposure. Moreover, intervention group experiments will be designed to introduce GDQ treatment following PM_2.5_ exposure, in order to further elucidate the temporal dynamics of GDQ’s efficacy and to determine the optimal timing for its administration. Our current selection of inflammatory biomarkers and detection methods is not sufficiently comprehensive. In future studies, we plan to include additional biomarkers such as sICAM-1, HMGB1, and P65, and apply techniques like flow cytometry, Western blotting, and PCR to further substantiate our conclusions.

## 5 Conclusion

In this study, we investigated the potential therapeutic effects of GDQ in alleviating PM2.5-induced chronic airway inflammation. Our results demonstrated that GDQ significantly reduced pathological lung tissue damage, attenuated inflammation, and improved lung function in a rat model exposed to PM2.5. The study also explored the mechanisms underlying GDQ’s effects, revealing its ability to modulate the lung microbiota and restore metabolic balance. Specifically, GDQ treatment was found to reduce the diversity and richness of the altered lung microbiota induced by PM2.5 exposure, while metabolomic analysis identified key metabolic pathways, including phospholipase D signaling, cytochrome P450-mediated metabolism of xenobiotics, and glutathione metabolism, as potential targets of GDQ’s action. Additionally, GDQ was shown to regulate the balance between pro-inflammatory TH17 cells and immune-regulatory Treg cells, suggesting an important role in modulating immune responses. Overall, this study provides new insights into the therapeutic potential of GDQ for protecting against lung injury caused by PM2.5 exposure and offers a promising approach for managing air pollution-induced chronic airway diseases.

## Data Availability

The original contributions presented in this study are included in this article/Supplementary material, further inquiries can be directed to the corresponding authors.

## Footnotes

1
https://www.mzcloud.org/
