# Supplementary figures and images for: Gan-du-qing attenuates PM2.5-induced Chronic Airway Inflammation via regulating the pulmonary microbiota and metabolic profiles
Source: Front Med (Lausanne). 2025 Sep 10;12:1560225. doi: 10.3389/fmed.2025.1560225 (PMC12457115; doi:10.3389/fmed.2025.1560225)

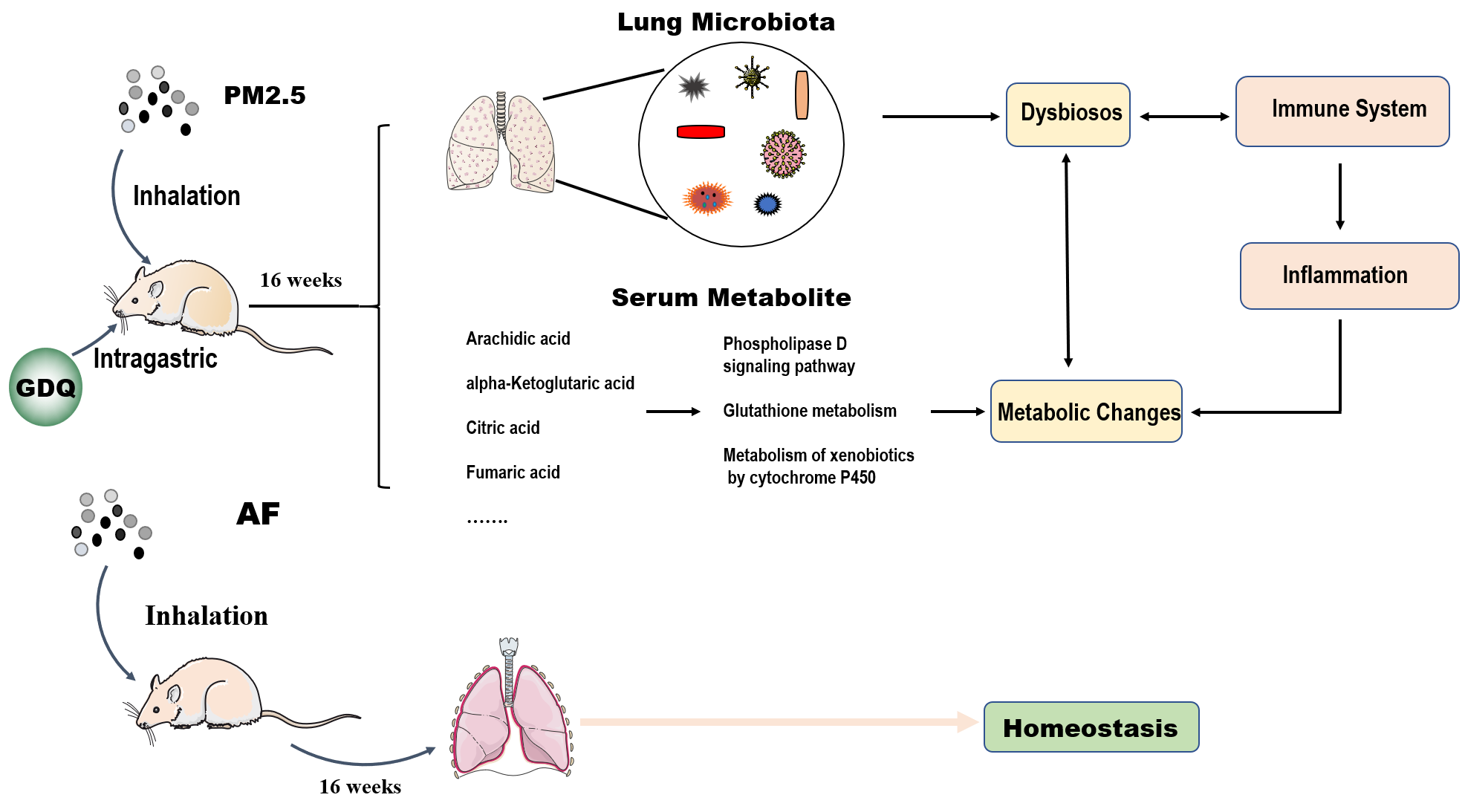

Supplement: Supplementary file 1 [file Image_1.tif]

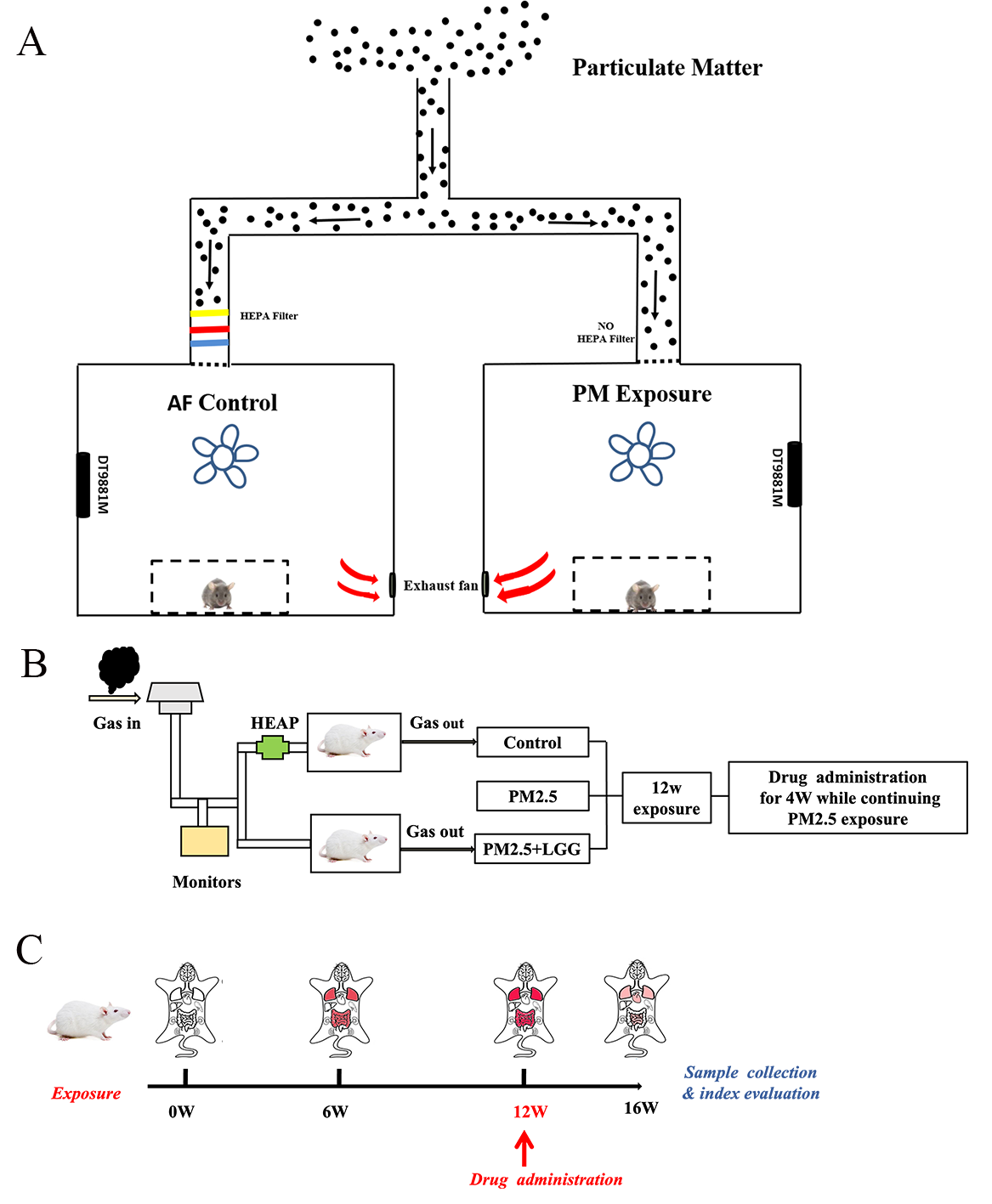

Supplement: Supplementary file 2 [file Image_2.tif]
